# Supplementary material for: Immune suppression in the early stage of COVID-19 disease
Source: Nat Commun. 2020 Nov 17;11:5859. doi: 10.1038/s41467-020-19706-9 (PMC7673112; doi:10.1038/s41467-020-19706-9)
Supplement: Supplementary file 1 — Supplementary Information [file 41467_2020_19706_MOESM1_ESM.pdf]

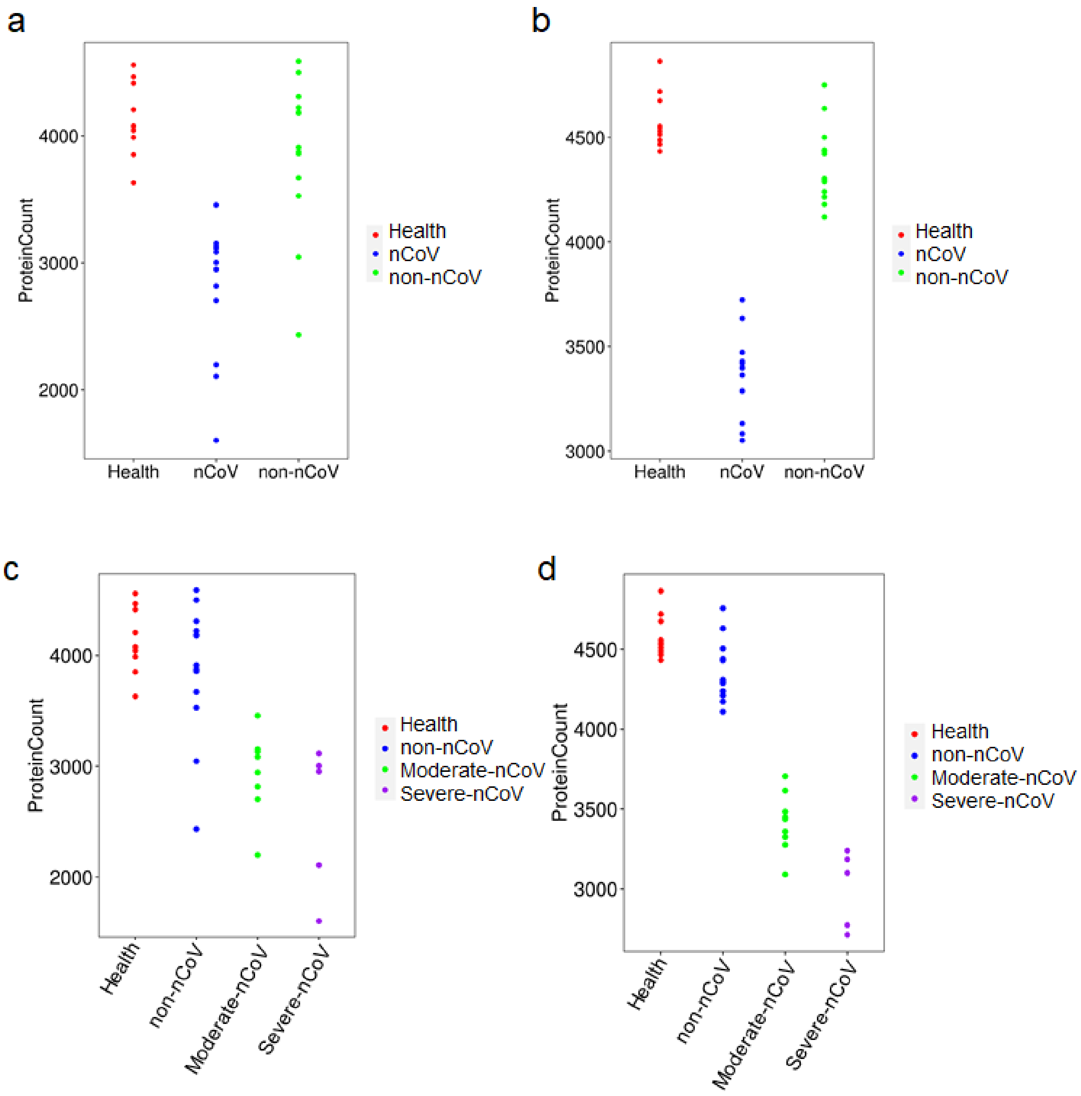

Supplementary Figure 1. Distribution of the number of identified proteins in three groups before and after imputing the missing data.

(a. b) Distribution map of the number of protein identified (a) before and (b) after data filling using a Double Boundary Bayes (DBB) Impute method in healthy control group (color red with "Health"); COVID-19 patient group (color blue with "nCoV"); and non-COVID-19 lung infection patient group (color green with "non-nCoV") The X-axis represents each sample group; the Y-axis represents the number of expressed proteins detected. Each point represents one sample. We observed a dramatic decreasing of total protein counts in COVID-19 patient group compare to healthy control and non-COVID-19 lung infection patient group.

(c. d) Distribution map of the number of protein identified (c) before and (d) after data filling using a Double Boundary Bayes (DBB) Impute method in healthy control group (color red with "Health"); non-COVID-19 patient group (color blue with "non-nCoV"); and two sub grouped COVID-19 patients (color green with "moderate-nCoV", color purple with "severe-nCoV") The X-axis represents each sample group, the Y-axis represents the number of expressed proteins detected. Each point represents one sample.

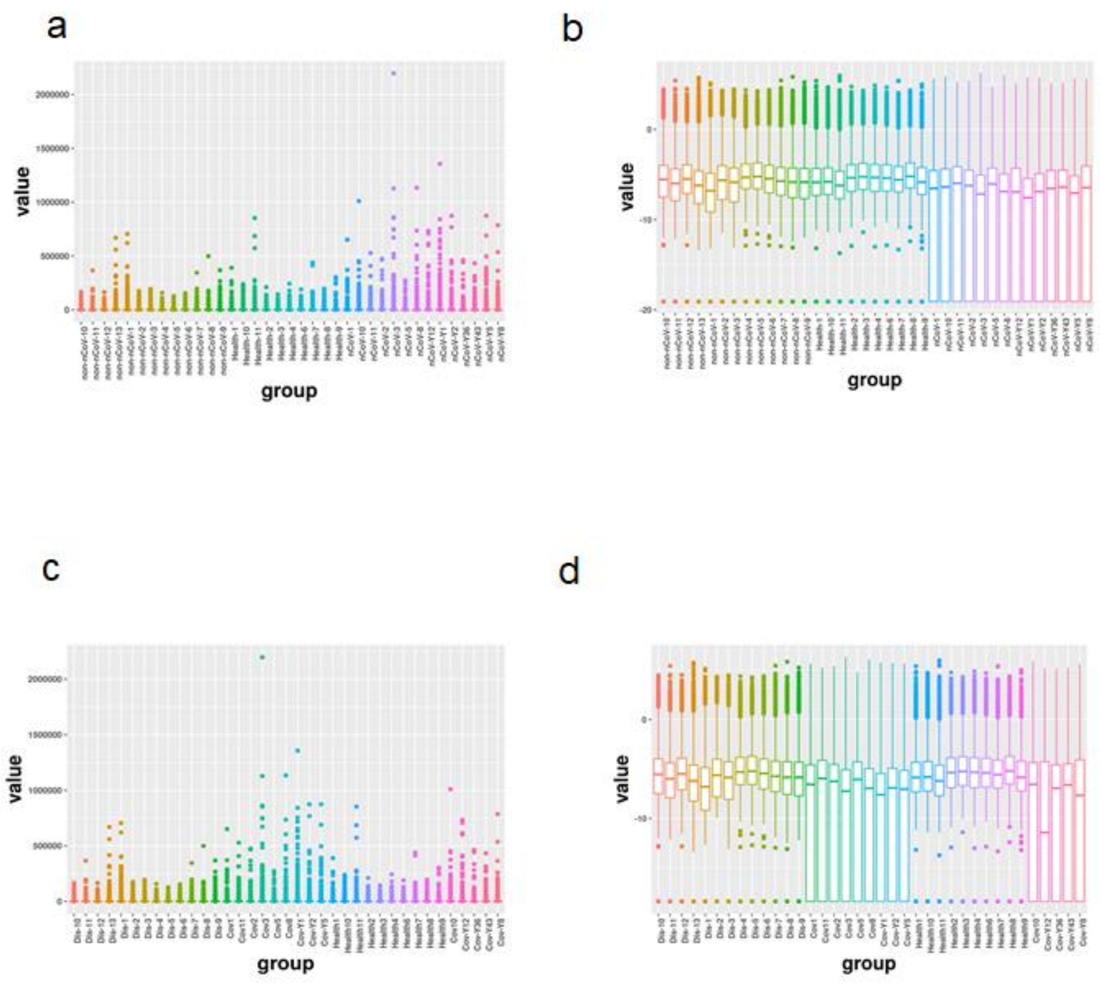

Supplementary Figure 2. Distribution map of data in each sample.

(a. b) Data of healthy control group, COVID-19 patient group and non-COVID-19 lung infection patient group before standardization (a) and after standardization (b).

(c. d) Data of healthy control group, COVID-19 patient group (Moderate type), COVID-19 patient group (Severe type) and non-COVID-19 lung infection patient group before standardization (c) and after standardization (d).

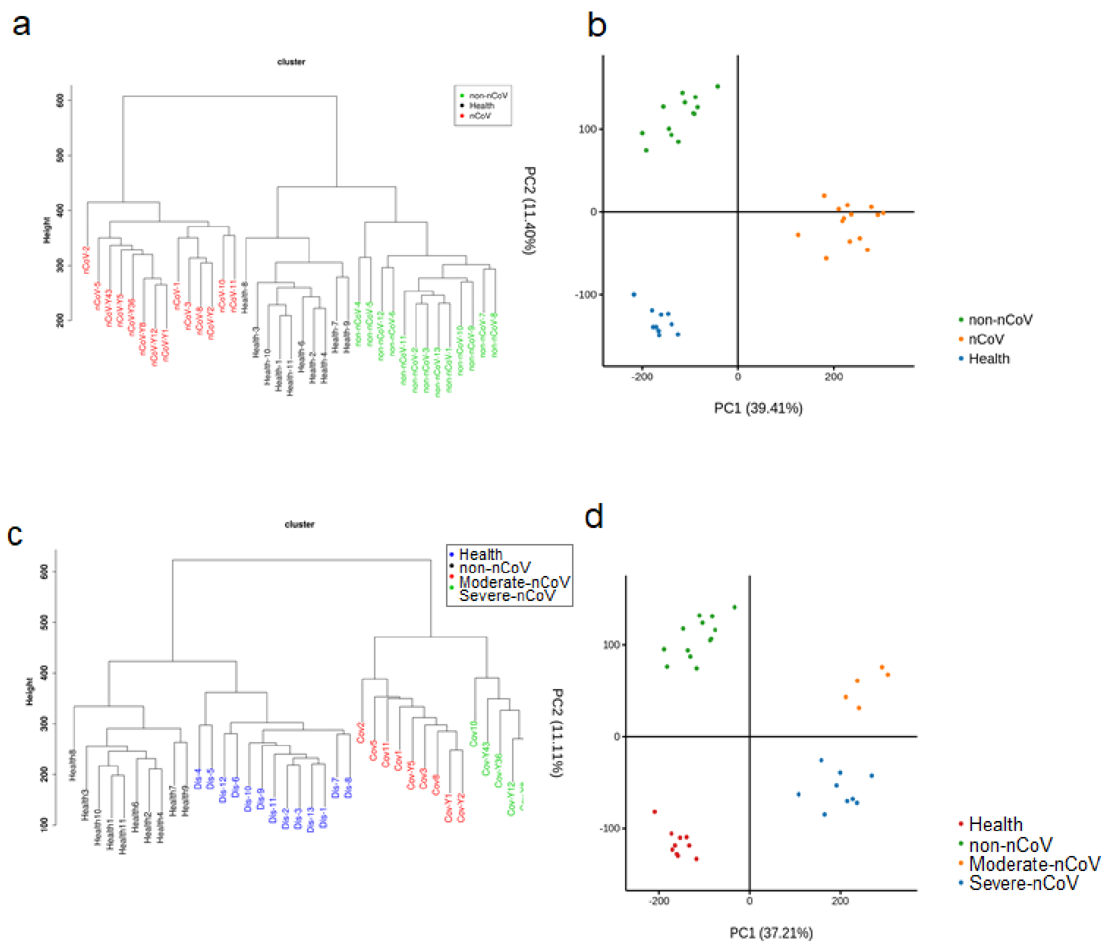

Supplementary Figure 3. Inter-group difference analysis.

(a. b) Cluster (a) and Principal Components Analysis (PCA) (b) showed the inter-group difference after standardization. Blue color with “health” represents healthy control group; orange color with “nCoV” represents COVID-19 patient group; green color with “non-nCoV” represents non-COVID-19 lung infection patient group. A clear stratification shows that the three groups can be well distinguished.

(c. d) Cluster (c) and Principal Components Analysis (PCA) (d) showed the inter-group difference after standardization. Red color with “health” represents healthy control group; orange color with “moderate-nCoV” represents moderate stage of COVID-19 patient; blue color with “severe-nCoV” represents severe stage of COVID-19 patient; green color with “non-nCoV” represents non-COVID-19 lung infection patient group. A clear stratification shows that the four groups can be well distinguished. nCOV group can be further divided into Moderate-nCoV and Severe-nCoV group, respectively.

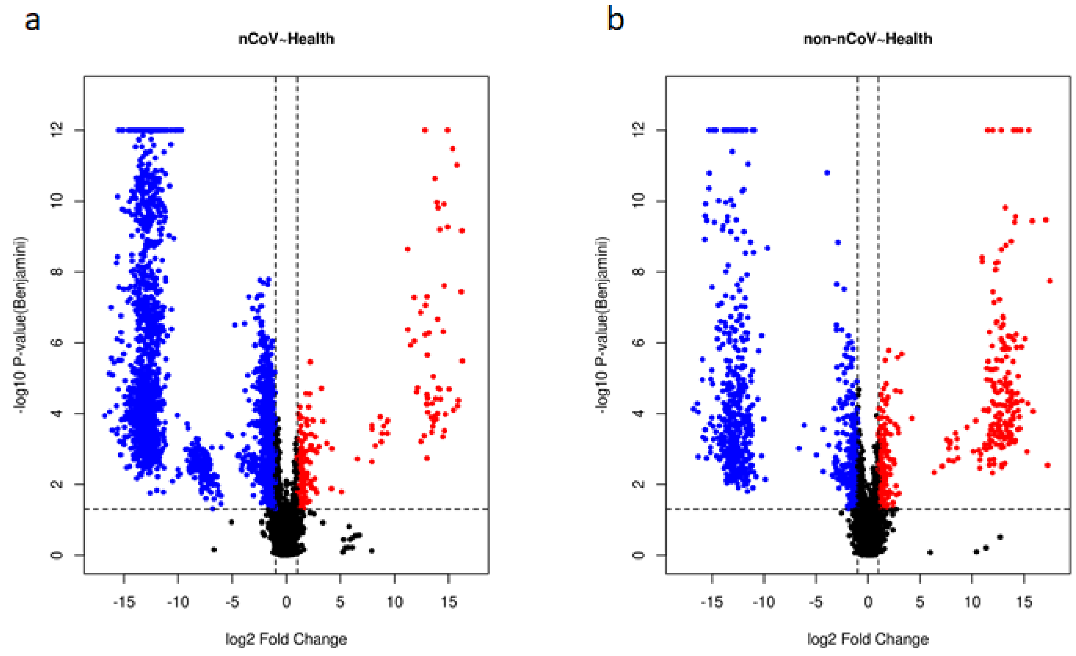

Supplementary Figure 4. Volcano plot of identified proteins in healthy control, COVID-19 patients and non-COVID-19 pneumonia patients.

Volcano plot of selected proteins (cut-off value of fold-change >2; P-value (t-test) <0.05) in the comparison between healthy control group vs COVID-19 lung infection patient group (A) or non-COVID-19 lung infection patient group (B). The y-axis represents the probability that the protein was differentially abundant. The red points or blue points sections with fold change >2 and $p < 0.05$ (t-test) represent proteins that were significantly increased or decreased in disease groups.

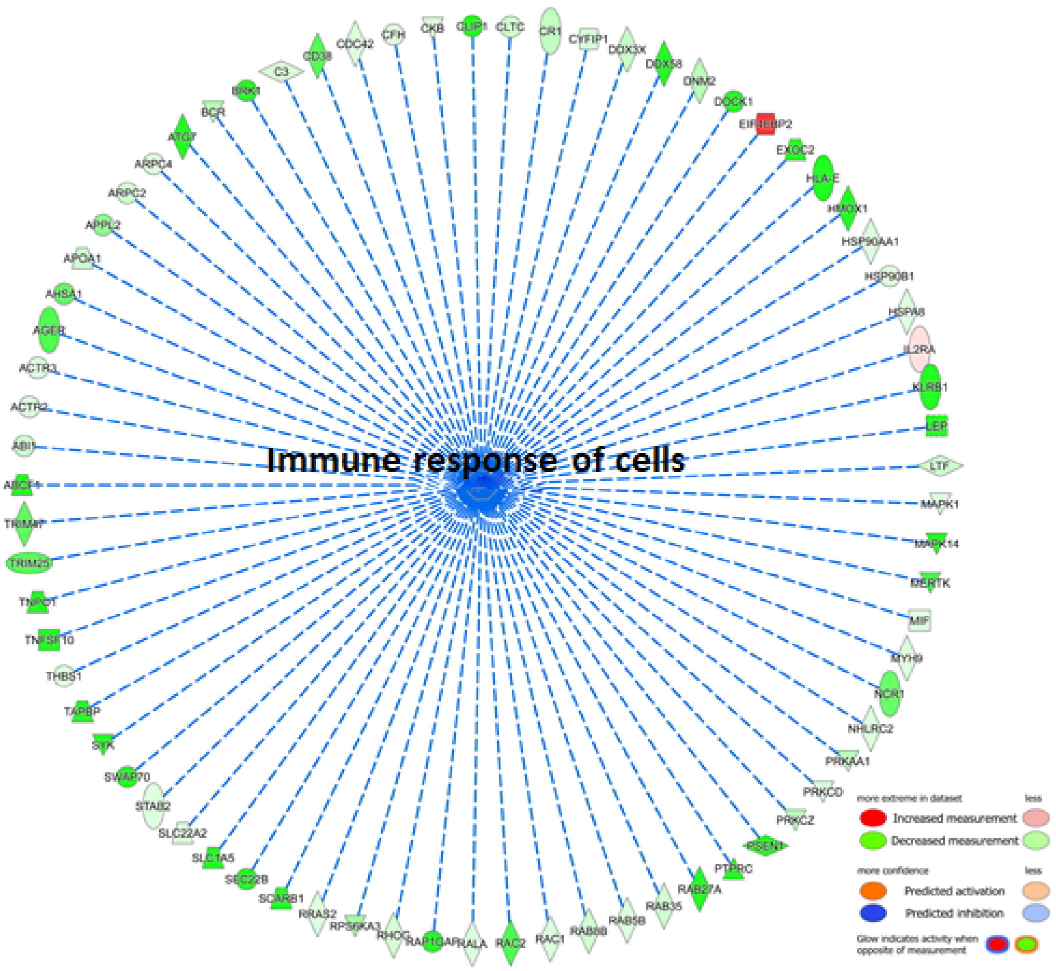

Supplementary Figure 5. Ingenuity pathway analysis (IPA) of 1986 proteins specific in COVID-19 patients showed the inhibited immune response of cells in patients with a z-score -3.169.

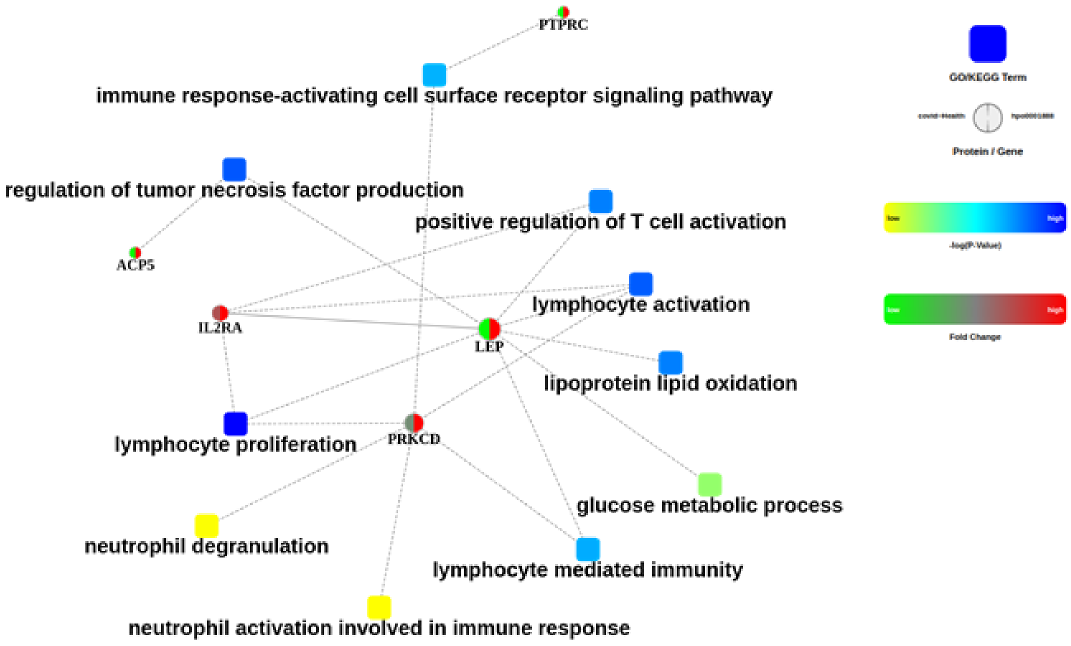

Supplementary Figure 6. Proteins related to lymphopenia decreased in COVID-19 patients. The network diagram of 5 proteins which are the overlaps of reported proteins in lymphopenia and 1986 proteins specific in COVID-19 patients. The circle representing the proteins is divided into two parts. The left part represents the proteins level in COVID-19 patients, color, red and gray color represents decreased, increased and no change respectively. The right part of the circle with red color represents the protein related to lymphopenia. Color bar from red to green represents the fold change of protein level from increasing to decreasing. The significance of the pathways represented by -log(P-Value) (Fisher's exact test) was shown by color scales with dark blue as most significance.

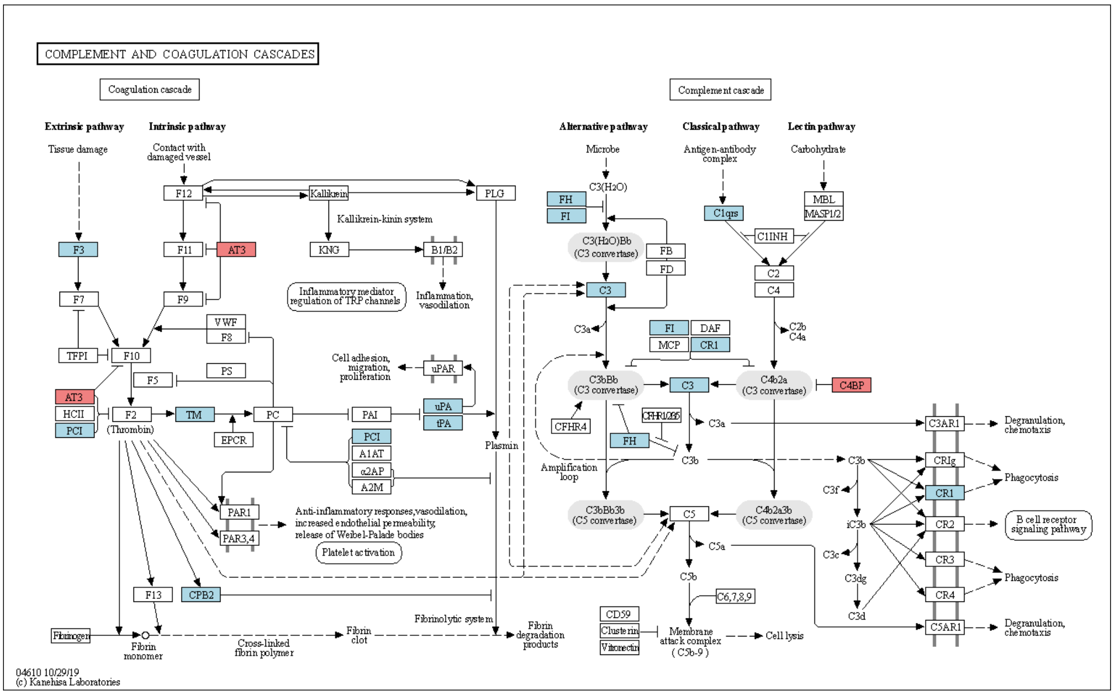

Supplementary Figure 7. KEGG pathway enrichment analysis of 1986 proteins specific in COVID-19 patients which indicate dysregulated complement and coagulation cascades. Protein selected in the analysis are fold change>2 or <0.5 and P value (t-test) <0.05. Blue color represents decreased protein level; red color represents increased protein level.

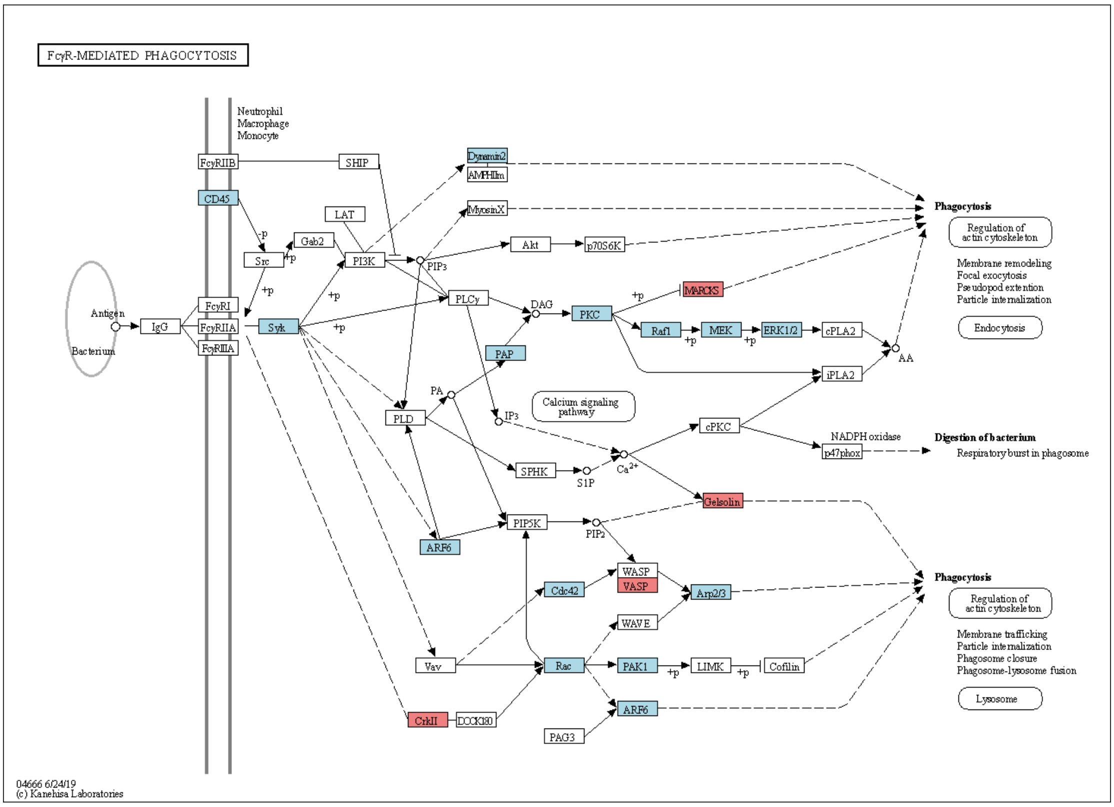

Supplementary Figure 8. KEGG pathway enrichment analysis of 1986 proteins specific in COVID-19 patients which indicate dysregulated FcγR mediated phagocytosis in COVID-19 patients. Protein selected in the analysis are fold change>2 or <0.5 and P value (t-test) <0.05. Blue color represents decreased protein level; red color represents increased protein level.

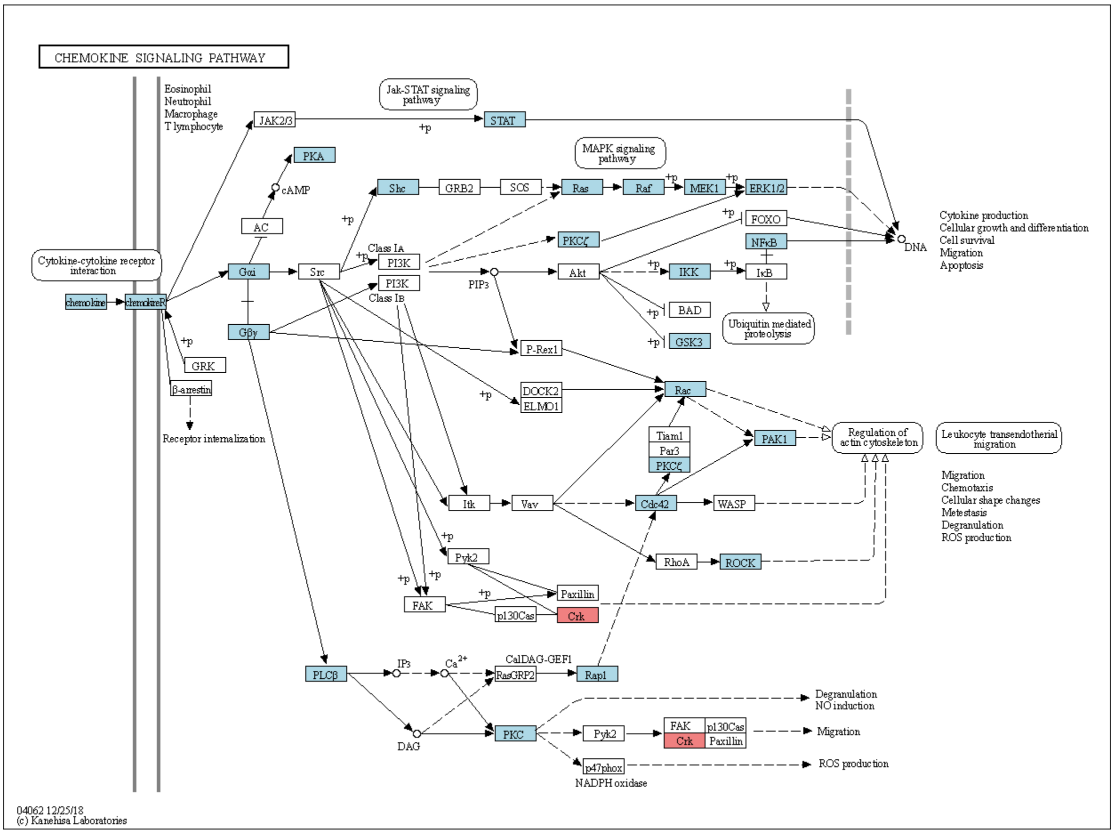

Supplementary Figure 9. KEGG pathway enrichment analysis of 1986 proteins specific in COVID-19 patients which indicate chemokine signaling pathway in COVID-19 patients. Protein selected in the analysis are fold change>2 or <0.5 and P value (t-test) <0.05. Blue color represents decreased protein level; red color represents increased protein level.

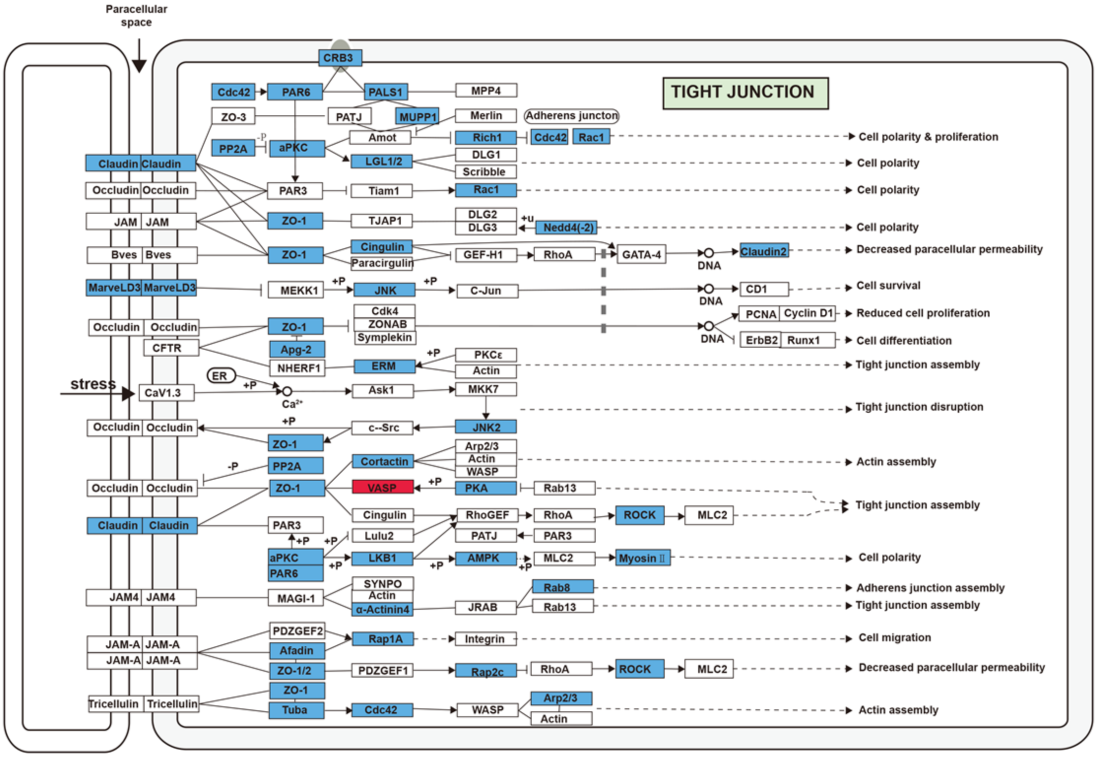

Supplementary Figure 10. KEGG pathway enrichment analysis of 1986 proteins specific in COVID-19 patients which suggest dysregulated tight junction in COVID-19 patients. Protein selected in the analysis are fold change>2 or <0.5 and P value (t-test) <0.05. Blue color represents decreased protein level; red color represents increased protein level.

a.

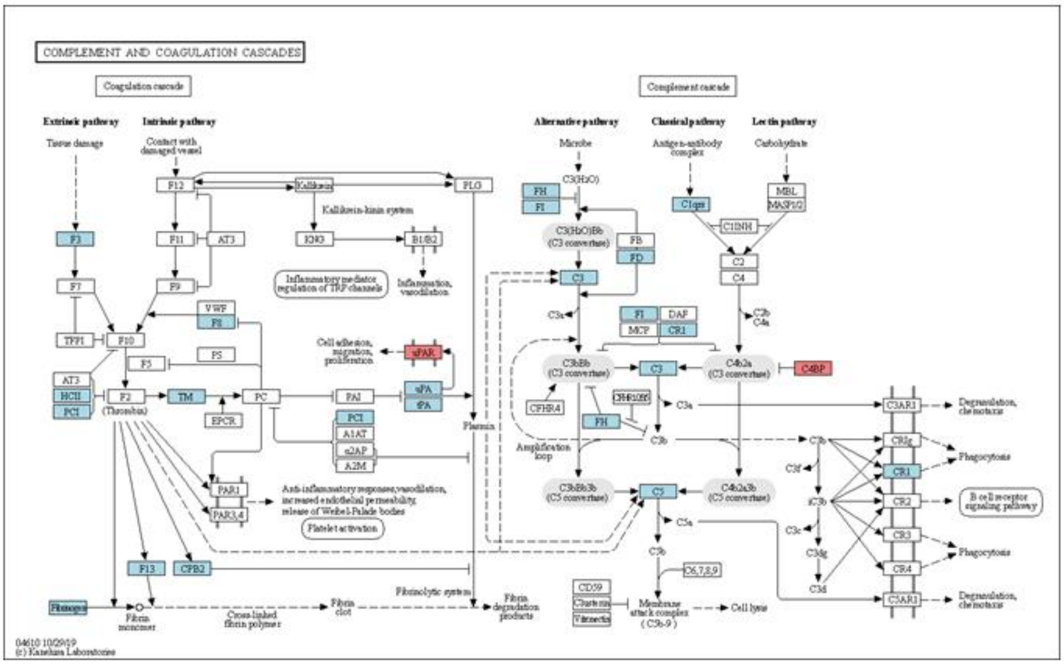

b.

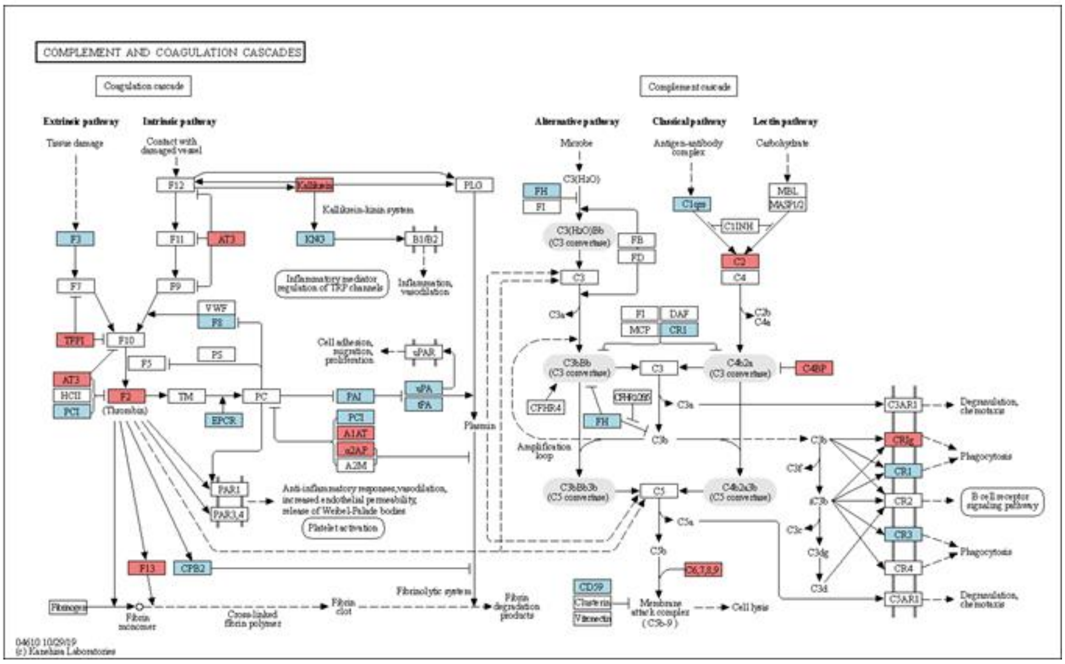

Supplementary Figure 11. KEGG pathway enrichment analysis of differentially expressed proteins in complement and coagulation cascades.

(a) KEGG pathway enrichment analysis of differentially expressed proteins in complement and coagulation cascades comparing moderate COVID-19 patients to health control. Protein selected in the analysis are fold change>2 or <0.5 and P value (t-test) <0.05. Blue color represents decreased protein level. Red color represents increased protein level.

(b) KEGG pathway enrichment analysis of differentially expressed proteins comparing severe COVID-19 patients to moderate COVID-19 patients, which indicate upregulated complement and coagulation cascades in the late stage of COVID-19 disease. Protein selected in the analysis are

fold change>2 or <0.5 and P value (t-test) <0.05. Blue color represents decreased protein level. Red color represents increased protein level.

a.

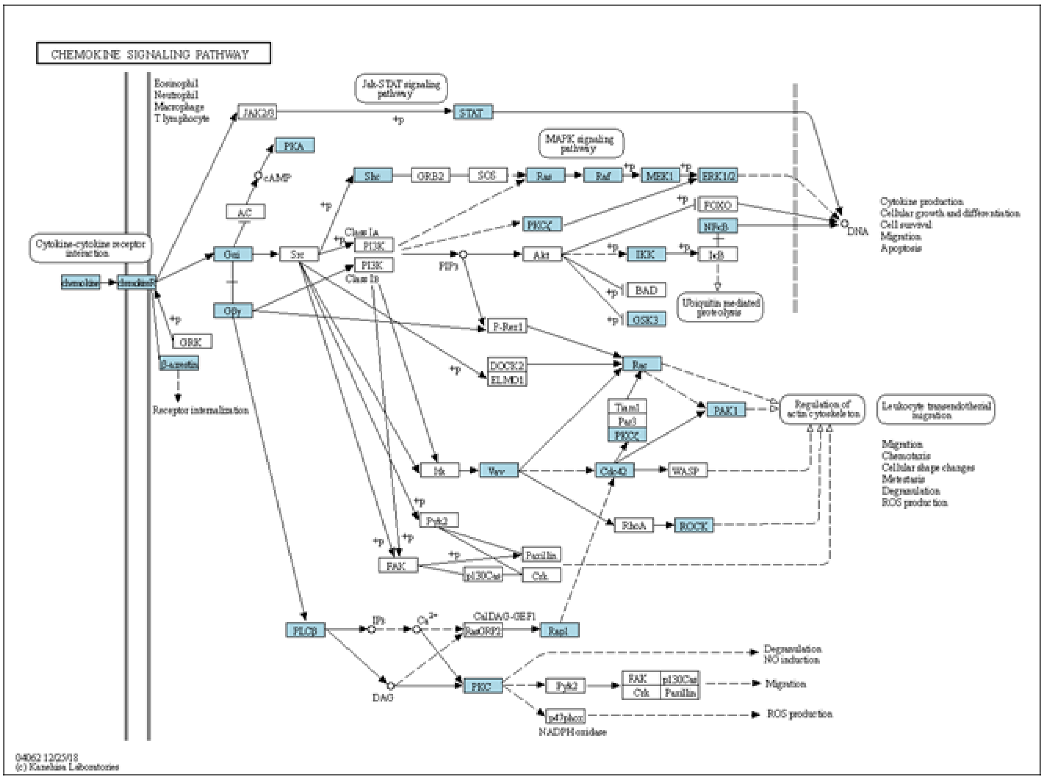

b.

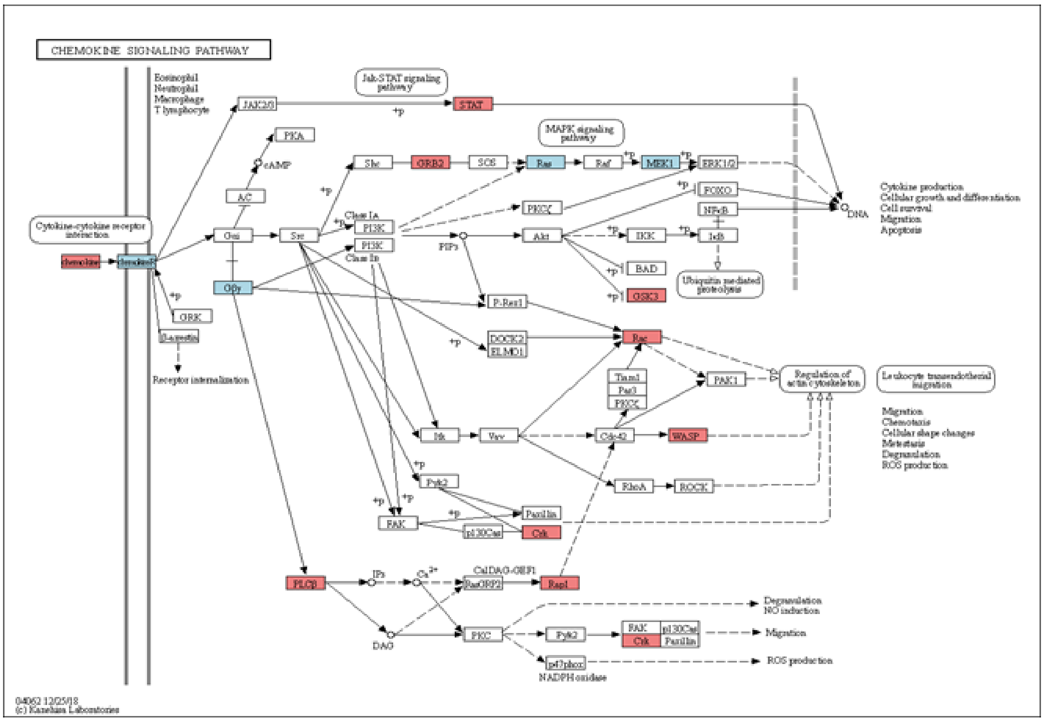

Supplementary Figure 12. KEGG pathway enrichment analysis of differentially expressed proteins in chemokine signaling pathway.

(a) KEGG pathway enrichment analysis of differentially expressed proteins in chemokine signaling pathway comparing moderate COVID-19 patients to health control. Protein selected in the analysis are fold change>2 or <0.5 and P value (t-test) <0.05. Blue color represents decreased

protein level. Red color represents increased protein level.

(b) KEGG pathway enrichment analysis of differentially expressed proteins comparing severe COVID-19 patients to moderate COVID-19 patients, which indicate upregulated chemokine signaling pathway in the late stage of COVID-19 disease. Protein selected in the analysis are fold change>2 or <0.5 and P value (t-test) <0.05. Blue color represents decreased protein level. Red color represents increased protein level.

a.

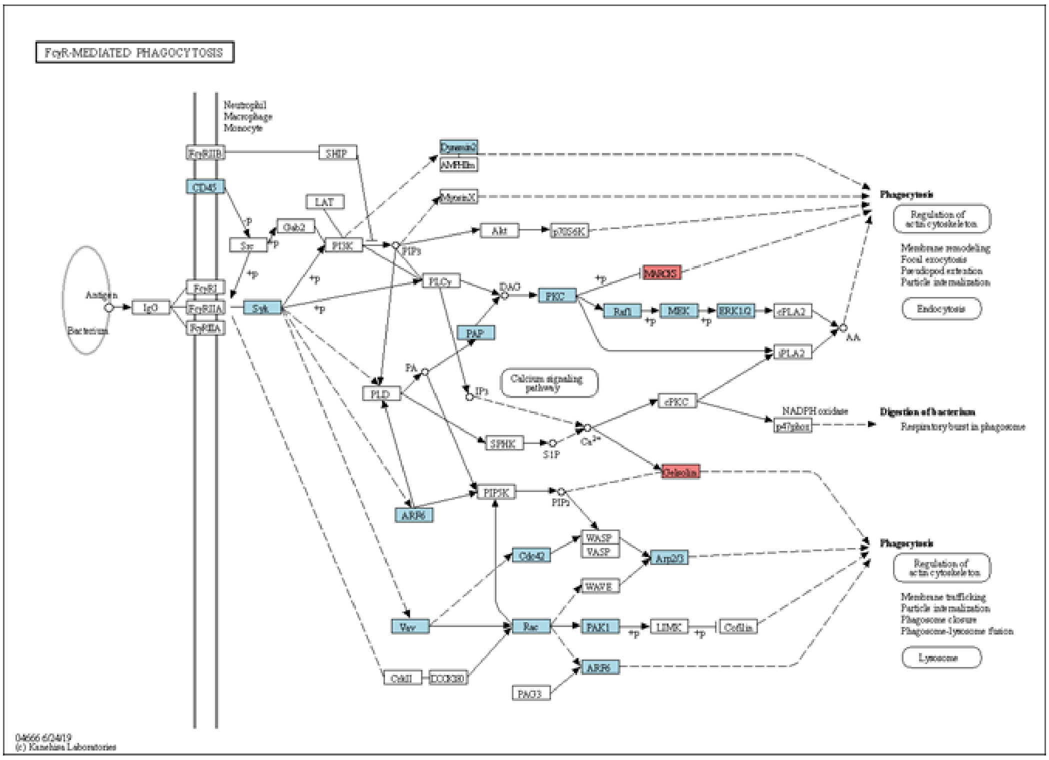

b.

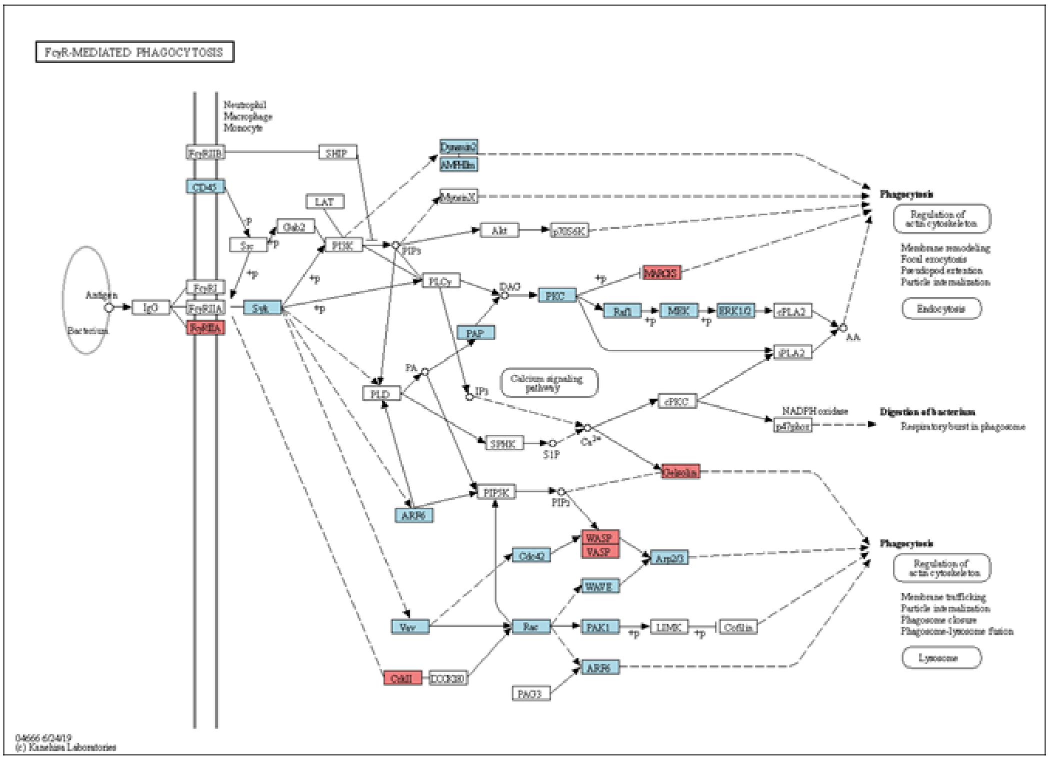

Supplementary Figure 13. KEGG pathway enrichment analysis of differentially expressed proteins in FcγR mediated phagocytosis.

(a) KEGG pathway enrichment analysis of differentially expressed proteins in FcγR mediated phagocytosis comparing moderate COVID-19 patients to health control. Protein selected in the analysis are fold change>2 or <0.5 and P value (t-test) <0.05. Blue color represents decreased protein level. Red color represents increased protein level.

(b) KEGG pathway enrichment analysis of differentially expressed proteins comparing severe COVID-19 patients to moderate COVID-19 patients, which indicate upregulated FcγR mediated phagocytosis in the late stage of COVID-19 disease. Protein selected in the analysis are fold change>2 or <0.5 and P value (t-test) <0.05. Blue color represents decreased protein level. Red color represents increased protein level.

Supplementary Table 1. Demographic information of patients enrolled in the study.

Group	ID	No	Age range	Gender	
Healthy Volunteers	Health-1	1	26-56	Male	
Healthy Volunteers	Health-2	2		Male	
Healthy Volunteers	Health-3	3		Male	
Healthy Volunteers	Health-4	4		Male	
Healthy Volunteers	Health-5	5		Male	
Healthy Volunteers	Health-6	6		Female	
Healthy Volunteers	Health-7	7		Male	
Healthy Volunteers	Health-8	8		Female	
Healthy Volunteers	Health-9	9		Female	
Healthy Volunteers	Health-10	10		Male	
Covid-19 patients	Cov-1	11	30-77	Female	moderate
Covid-19 patients	Cov-2	12		Male	moderate
Covid-19 patients	Cov-3	13		Male	moderate
Covid-19 patients	Cov-5	14		Female	moderate
Covid-19 patients	Cov-8	15		Male	moderate
Covid-19 patients	Cov-11	16		Female	moderate
Covid-19 patients	Cov-Y1	17		Female	moderate
Covid-19 patients	Cov-Y2	18		Female	moderate
Covid-19 patients	Cov-Y5	19		Male	moderate
Covid-19 patients	Cov-Y36	20		Male	severe
Covid-19 patients	Cov-10	21		Female	severe
Covid-19 patients	Cov-Y43	22		Female	severe
Covid-19 patients	Cov-Y8	23		Female	severe

Covid-19 patients	Cov-Y12	24		Male	severe
Non-COVID-19 pneumonia patients	Dis-1	25		Female	
Non-COVID-19 pneumonia patients	Dis-9	26		Male	
Non-COVID-19 pneumonia patients	Dis-3	27		Female	
Non-COVID-19 pneumonia patients	Dis-10	28		Female	
Non-COVID-19 pneumonia patients	Dis-8	29		Female	
Non-COVID-19 pneumonia patients	Dis-13	30		Male	
Non-COVID-19 pneumonia patients	Dis-6	31	25-78	Female	
Non-COVID-19 pneumonia patients	Dis-7	32		Male	
Non-COVID-19 pneumonia patients	Dis-5	33		Female	
Non-COVID-19 pneumonia patients	Dis-2	34		Female	
Non-COVID-19 pneumonia patients	Dis-12	35		Female	
Non-COVID-19 pneumonia patients	Dis-11	36		Female	
Non-COVID-19 pneumonia patients	Dis-4	37		Female	

Supplementary Table 2. Clinical Characteristics of Covid-19 patients.

**No.**	11	12	13	14	15	16	17	18	19	20	21	22	23	24
**Physical Examination**														
body tempearture(degree)	36.1	38.7	37	36.9	38	37.3	37.7	37.7	37.2	37.5	36.3	36.5	36	37
pulse (beats/min)	76	80	99	80	86	80	78	102	90	80	80	82	80	80
respiratory rate (beats/min)	19	20	20	19	21	18	20	20	21	20	18	21	17	20
oxygen saturation %	99	98	97	98	96	99	98	99	95.6	98.5	98	98.9	98	99
**Clinical Laboratory and Imaging Test**														
white blood cells (x10^9/L)	4.61	6.8	3.3	6.3	4.01	4.09	2.87	4.68	3.92	4.91	3.78	2.76	5.24	3.6
neutrophils (x10^9/L)	1.98	4.61	1.21	3.35	4.37	3.11	1.77	1.95	2.4	3.43	1.73	1.76	2.44	0.61
monocytes(x10^9/L)	0.44	0.24	0.4	0.54	1.22	0.24	0.17	0.4	0.27	0.38	0.35	0.17	1.51	0.54
Neutrophil percentage %	42.9	83.7	66.6	53.2	68.04	76.1	67.24	41.47	71.14	39.34	69.34	58.44	46.6	41.7
red blood cell(x10^12/L)	3.97	4.89	5.38	6.15	4.74	3.31	4.14	5.23	4.7	4.98	5.23	5.08	4.61	5.25
hemoglobin (g/L)	129	137	170	127	156	105	126	157	156	159	157	115	140	156
platelet(x10^9/L)	209	246	134	237	240	156	206	173	154	224	217	158	178	182
troponin I (ng/mL)	<0.1	<0.1	<0.1	<0.1	N/A	0.11	<0.1	<0.1	0.037	0.1	N/A	0.013	N/A	<0.1
creatine kinase -MB isoenzyme (ng/mL)	1.11	0.99	1.28	1.88	1.57	2.33	0.27	1.89	0.66	0.9	N/A	0.19	1.54	1.66
Myoglobin MB (ng/mL)	14.59	10	11	12	37	11.6	34	14	78	53	N/A	31	N/A	10
Procalcitonin :& LT(ng/mL)	<0.1	0.05	< 0.05	< 0.05	< 0.05	<0.1	0.12	0.05	0.17	0.11	< 0.05	0.13	< 0.05	0.05
lymphocyte(x10^9/L)	N/A	0.6	0.7	N/A	N/A	N/A	0.77	N/A	1.05	0.94	N/A	0.84	N/A	N/A

lymphocyte percentage %	N/A	8.6	22	N/A	N/A	15.6	N/A	N/A	N/A	N/A	N/A	N/A	N/A	N/A
C-Reactive protein (mg/L)	N/A	24.7	3.57	0.57	N/A	N/A	13	N/A	52.7	22	N/A	22	N/A	N/A
eosinophil percentage %	N/A	0.4	N/A	N/A	N/A	2.4	N/A	N/A	N/A	N/A	N/A	N/A	N/A	N/A
serum creatinine （umol/L)	N/A	N/A	N/A	N/A	N/A	N/A	71	N/A	51	86	N/A	44	N/A	N/A
eGFR (ml/min/1.73m^2)	N/A	N/A	N/A	N/A	N/A	N/A	83.2	N/A	108.8	61.1	N/A	100.3	N/A	N/A
urinary protein	N/A	N/A	N/A	N/A	N/A	N/A	+	neg	neg	neg	neg	neg	neg	+
Chest CT	Patchy shadows with irregular shape and uneven density can be seen in the right lung.	No abnormality	Inflammation of both lungs	Left lower pneumonia (improved) Right lower lung mass transformation (new)	Ground glass image of upper lobe of left lung	The lower lobe of right lung has a little ground glass shadow	Multiple ground glass opacity on both lungs	Inflammation of the lower lobe of both lungs	Multiple ground glass opacity and cord shadow on both lungs	Multiple patchy density shadows in both lungs，pulmonary bullae on the right lung	Subpleural infectious lesions in the lower lobe of both lungs	Multiple ground glass opacity, patchy density shadows and cord shadow on both lungs	Two lung ground glass density shadow	Two pulmonary septal emphysema, a few chronic inflammatory cords in both lungs
