# Supplementary material for: Immune suppression in the early stage of COVID-19 disease
Source: Nat Commun. 2020 Nov 17;11:5859. doi: 10.1038/s41467-020-19706-9 (PMC7673112; doi:10.1038/s41467-020-19706-9)
Supplement: Supplementary file 3 — Descriptions of Additional Supplementary Files [file 41467_2020_19706_MOESM3_ESM.docx]

**Description of Supplementary Files**

**File Name:** **Supplementary Data 1**

**Description:** Raw data from mass spectrometry-based, data-independent acquisition (DIA) quantitative proteomic analysis of urine samples from healthy donors, COVID-19 patients, and non COVID-19 pneumonia patients.

**File Name:** **Supplementary Data 2**

**Description:** Double Boundary Bayes (DBB) imputation of the raw data from supplementary data 1 grouped into healthy donors, COVID-19 patiens and non-COVID-19 pneumonia patients.

**File Name:** **Supplementary Data 3**

**Description:** Normalization of Double Boundary Bayes (DBB) imputated data from supplementary data 2.

**File Name:** **Supplementary Data 4**

**Description:** Differential protein abundance in COVID-19 patients compared to healthy donors.

**File Name:** **Supplementary Data 5**

**Description:** Differential protein abundance in COVID-19 patients compared to healthy donors.

**File Name:** **Supplementary Data 6**

**Description:** 145 protein list in Figure 2b.

**File Name:** **Supplementary Data 7**

**Description:** The significantly changed 1986 proteins specific in COVID-19 patients.

**File Name:** **Supplementary Data 8**

**Description:** Double Boundary Bayes (DBB) imputation of the raw data from supplementary data 1 grouped into healthy donors, moderate COVID-19 patiens, sever COVID-19 patients and non-COVID-19 pneumonia patients.

**File Name:** **Supplementary Data 9**

**Description:** Normalization of Double Boundary Bayes (DBB) imputated data from supplementary data 9.

**File Name:** **Supplementary Data 10**

**Description:** The significantly changed proteins comparing moderate subgroup and sever subgroup.

**File Name:** **Supplementary Data 11**

**Description:** The significantly changed proteins comparing moderate subgroup and healthy donor.
