# Supplementary material for: Immune suppression in the early stage of COVID-19 disease
Source: Nat Commun. 2020 Nov 17;11:5859. doi: 10.1038/s41467-020-19706-9 (PMC7673112; doi:10.1038/s41467-020-19706-9)
Supplement: Supplementary file 15 — Reporting Summary [file 41467_2020_19706_MOESM15_ESM.pdf]

nature research

Corresponding author(s): Wenjie Tan, Yang Chen, George, Fu Gao, Catherine CL Wong

Last updated by author(s): Sep 28, 2020

Reporting Summary

Nature Research wishes to improve the reproducibility of the work that we publish. This form provides structure for consistency and transparency in reporting. For further information on Nature Research policies, see our Editorial Policies and the Editorial Policy Checklist.

Statistics

For all statistical analyses, confirm that the following items are present in the figure legend, table legend, main text, or Methods section.

n/a	Confirmed	
☐	☒	The exact sample size (*n*) for each experimental group/condition, given as a discrete number and unit of measurement
☐	☒	A statement on whether measurements were taken from distinct samples or whether the same sample was measured repeatedly
☐	☒	The statistical test(s) used AND whether they are one- or two-sided<br>*Only common tests should be described solely by name; describe more complex techniques in the Methods section.*
☒	☐	A description of all covariates tested
☐	☒	A description of any assumptions or corrections, such as tests of normality and adjustment for multiple comparisons
☐	☒	A full description of the statistical parameters including central tendency (e.g. means) or other basic estimates (e.g. regression coefficient) AND variation (e.g. standard deviation) or associated estimates of uncertainty (e.g. confidence intervals)
☐	☒	For null hypothesis testing, the test statistic (e.g. *F*, *t*, *r*) with confidence intervals, effect sizes, degrees of freedom and *P* value noted<br>*Give P values as exact values whenever suitable.*
☐	☒	For Bayesian analysis, information on the choice of priors and Markov chain Monte Carlo settings
☐	☒	For hierarchical and complex designs, identification of the appropriate level for tests and full reporting of outcomes
☐	☒	Estimates of effect sizes (e.g. Cohen's *d*, Pearson's *r*), indicating how they were calculated

Our web collection on statistics for biologists contains articles on many of the points above.

Software and code

Policy information about availability of computer code

Data collection	The mass spectrometer was operated in data-dependent mode for the ion mobility enhanced spectral library generation. We set the accumulation and ramp time was 100 ms each and recorded mass spectra in the range from m/z 100–1700 in positive electrospray mode. The ion mobility was scanned from 0.6 to 1.6Vs/cm2. The overall acquisition cycle of 1.16s comprised one full TIMS-MS scan and 10 PASEF MS/MS scans. When perform data-independent acquisition, we define quadrupole isolation windows as a function of the TIMS scan time to achieve seamless and synchronous ramps for all applied voltages. We defined up to 8 windows for single 100 ms TIMS scans according to the m/z-ion mobility plane. During PASEF MSMS scanning, the collision energy was ramped linearly as a function of the mobility from 59 eV at 1/K0=1.6 Vs cm-2 to 20 eV at 1/K0=0.6 Vs cm-2.<br>Raw files were processed using a developmental version of Spectronaut (v14.0.200409.43655, Biognosys). The ion mobility enhanced library was generated from dda-PASEF raw data using Spectronaut's Pulsar database search engine with 1% FDR control at PSM, peptide and protein level. Carbamidomethyl (C) was set as fixed modifications, and Oxidation (M) and Acetyl (Protein N-term) were set as variable modifications. For the subsequent targeted analysis of diaPASEF data, DIA files were processed using Spectronaut with default settings, but the correction factor of XIC IM extraction window set to 0.8 instead of 1.0. Q-values at precusor and protein level were set to less than 1%.
Data analysis	To impute the proteomic data, we first used locally-weighted polynomial regression (lowess in R version 3.6.3) to compute the local polynomial fit for protein number and protein detecting rate in each stage(time point). Two boundary thresholds, 0.15 and 0.5, were used to separate the data into 3 parts. When a protein detecting rate is lower than 0.15, it's probably the detected value is due to a technical error. For these proteins, no imputation was applied. When a protein detecting rate is above 0.5, the missing value was probably due to the detection accuracy limitation of the LC/MS. In this case, the missing value was replaced with median value. When a protein detecting rate is between 0.15 and 0.5, it's probably the protein expression is unstable for detection.<br>In this case, we first calculated the missing probability of a protein using bayes theory, missp=PA*(PBA/((PBA*PA)+(0.05*(1-PA))))<br>where, PBA: group missing rate(PBA), PA: total missing rate(PA) of each protein.<br>Then we determined the predicted imputation number(IN) of each protein in each group,<br>IN =(1-missp) * Mi

Where, Mi: undetected sample number of a protein in group i
And finally the random method was used to determine the samples to be imputated. The imputation value was then defined by,
IV = min(Mi/2, IN)

Imputed data were then normalized using LogNorm algorithm. PCA (muma v1.4 package, https://www.rdocumentation.org/packages/muma) and fastcluster v.1.1 (https://www.rdocumentation.org/packages/fastcluster/versions/1.1.25/) using euclidean distance was used to perform the clustering analysis of samples.
R package Genefilter (https://www.rdocumentation.org/packages/genefilter/versions/1.54.2) was used in calculation of the fold change values of proteins. Fold change of 2 and p value of 0.05 were used to filter differential expression proteins.
Mfuzz Version 2.46.0 (https://www.bioconductor.org/packages/release/bioc/html/Mfuzz.html) was used to detect different sub-clustering models of gene expression among groups. R version 3.6.3 was used to implement fisher exact test. String version 11 was used for protein-protein interaction network analysis.
Venn diagram, heatmap, and network visualization were performed using the ggplot2 packages and Cytoscape version 3.5.1 implemented in the omicsbean workbench. Ingenuity Pathway Analysis was performed to explore the downstream effect in significant regulated proteins dataset. The z-score algorithm was used to predict the activation state (either activated or inhibited) of biological process. If the z-score ≤ -2, the process is predicted to be statistically significantly inhibited.

For manuscripts utilizing custom algorithms or software that are central to the research but not yet described in published literature, software must be made available to editors and reviewers. We strongly encourage code deposition in a community repository (e.g. GitHub). See the Nature Research guidelines for submitting code & software for further information.

Data

Policy information about availability of data

All manuscripts must include a data availability statement. This statement should provide the following information, where applicable:
- Accession codes, unique identifiers, or web links for publicly available datasets
- A list of figures that have associated raw data
- A description of any restrictions on data availability

Reference FASTA files contain human UNIPROT database (only reviewed entries) (human 20,421 entries, downloaded July 2019) and SARS-CoV-2 Uniprot database which combined with SARS protein database (38 viral entries, April 2020). Latest GO database32 (https://www.ebi.ac.uk/QuickGO/) and KEGG pathway database33 (https://www.kegg.jp/kegg/pathway.html) were used for gene ontology and pathway enrichment analysis. The KEGG ligand database (https://www.kegg.jp/kegg/ligand.html) was used to obtain the compound and enzyme relationship. The experimental data that support the findings of this study have been deposited in iProX (integrated proteome resources) of ProteomeXchange with the accession code PXD020522. The data could be accessed from https://www.iprox.org/page/SSV024.html;url=1601217952947riX4 with Extraction password of 3Efy. The source data underlying Fig. 1c, Fig. 2ab, Fig. 2c, Fig. 3ac, Fig. 3b, Fig. 3d are provided as a Source Data file. Source data are provided with this paper.

Field-specific reporting

Please select the one below that is the best fit for your research. If you are not sure, read the appropriate sections before making your selection.

☒ Life sciences ☐ Behavioural & social sciences ☐ Ecological, evolutionary & environmental sciences

For a reference copy of the document with all sections, see nature.com/documents/nr-reporting-summary-flat.pdf

Life sciences study design

All studies must disclose on these points even when the disclosure is negative.

Sample size	We did not perform statistical analyses to predetermine sample sizes. The urine samples of COVID-19 patients are very precious, so the sample size of 14 was mainly determined by the access and availability of patient samples and materials. The sample sizes for other two groups were accordingly determined, which were 13 (non-COVID-19 pneumonia patients) and 10 (healthy people), respectively.
Data exclusions	No patient was excluded for the current study.
Replication	Data Independent Acquisition (DIA) technique was applied for quantitative proteomic analysis. Each patient sample is treated as a biological duplicate. A quality control (QC) sample of mixed-aliquots from each sample was applied every four samples run. The median coefficient of variation (CV) for quantitication was 18.6% on the protein level after median normalization.
Randomization	Randomization is not applicable in this study, as the patients were recruited retrospectively based on the clinical diagnosis and treatment guideline.
Blinding	For all the experiments, the investigators were blinded to group allocation, as well as data analysis. For the statistical analysis, no blinding was undertaken in order to deeply excavate the information contained in the datasets.

Reporting for specific materials, systems and methods

We require information from authors about some types of materials, experimental systems and methods used in many studies. Here, indicate whether each material, system or method listed is relevant to your study. If you are not sure if a list item applies to your research, read the appropriate section before selecting a response.

Materials & experimental systems

n/a	Involved in the study	
☒	☐	Antibodies
☒	☐	Eukaryotic cell lines
☒	☐	Palaeontology and archaeology
☒	☐	Animals and other organisms
☐	☒	Human research participants
☒	☐	Clinical data
☒	☐	Dual use research of concern

Methods

n/a	Involved in the study	
☒	☐	ChIP-seq
☒	☐	Flow cytometry
☒	☐	MRI-based neuroimaging

Human research participants

Policy information about studies involving human research participants

Population characteristics	For the Healthy cohort, the median age was 35.5 years, ranged from 26 years to 56 years, and 7 of 10 were male. For the COVID-19 cohort, the median age was 57 ranged from 30 to 77 years, and 6 of 14 were male. For Non-COVID-19 pneumonia patients, the median age was 31 ranged from 26 to 78 years, and 3 of 13 were male
Recruitment	1) COVID-19 Patients were diagnosed according to the Chinese Government Diagnosis and Treatment guideline (Trial 5th Version) (Medicine 2020). Non-COVID-19 Lung Infection patients were diagnosed with positive chest CT/MRI/X-ray images and negative RT-PCR results. 2) Patients were recruited from Beijing Youan Hospital, Capital Medical University and Chinese Center for Disease Control and Prevention. 3) Patients agreed to be enrolled.<br>Because samples from COVID-19 patients were hard to access, we enrolled as many patients as possible in this study. Samples from Non-COVID-19 pneumonia patients and healthy people were selected randomly from the clinical biological sample bank in the hospital. Therefore, no self-selection bias were present.
Ethics oversight	Ethics approval was exempted from institutional review board of the hospital since we collected and analyzed all data from the patients according to the policy issued by the National Health Commission of the People's Republic of China. Written informed consent was obtained from each participant.

Note that full information on the approval of the study protocol must also be provided in the manuscript.
